# Elevated serum Slit3 independently predicts disease activity and interstitial lung disease in rheumatoid arthritis

**DOI:** 10.1016/j.clinsp.2026.100935

**Published:** 2026-04-08

**Authors:** Xuepei Zhang, Maohua Shi, Dongmei Guo, Yangtao Yu, Hongwei Zhang, Guoqiang Chen

**Affiliations:** a Department of Rheumatology, First People's Hospital of Foshan, China; b Department of Rheumatology, Second People's Hospital of Foshan, China

**Keywords:** Rheumatoid arthritis, Slit guidance ligand 3, Inflammation, Interstitial lung disease

## Abstract

**Introduction:**

Rheumatoid Arthritis (RA) is a chronic inflammatory disease often linked to Interstitial Lung Disease (ILD). Slit guidance Ligand-3 (Slit3), a member of the axon guidance molecule superfamily, mediates a series of biological activities, including the regulation of the inflammatory response and fibrosis. This study explored Slit3’s clinical significance as a serum biomarker for RA-ILD.

**Methods:**

Between November 2022 and April 2024, patients diagnosed with RA were included in the Foshan First People's Hospital. The clinical data of patients were collected from hospital records, and the serum level of Slit3 was measured using an enzyme-linked immunosorbent assay. Logistic regression was employed to determine odds ratios for Slit3 and other indicators associated with the development of RA-ILD.

**Results:**

In total, 232 patients were included. Patients with RA in the high Slit3 subgroup were older, were more likely to be positive for rheumatoid factor and anti-cyclic citrullinated peptide antibody, and had higher levels of erythrocyte sedimentation rate and C-reactive protein. They also showed higher Disease Activity Scores (DAS28-CRP, DAS28-ESR, SDAI) as well as elevated pro-inflammatory cytokine interleukin-6. Notably, logistic regression analysis indicated that the elevated serum level of Slit3 was an independent predictor of RA-ILD.

**Conclusions:**

Elevated serum level of Slit3 was associated with higher disease activity and served as an independent risk factor of RA-ILD. Slit3 may hold promise as a useful serum biomarker and therapeutic target for RA-ILD.

## Highlights

• First clinical evidence linking serum Slit3 to both RA disease activity and RA-associated Interstitial Lung Disease (RA-ILD).
• Slit3 as an independent predictor of RA-ILD, alongside male gender, disease duration, and ACPA positivity.
• High Slit3 levels correlate with increased systemic inflammation (ESR, CRP, IL-6) and higher composite Disease Activity Scores (DAS28, SDAI).
• Weak-to-moderate correlation with HRCT lung involvement suggests Slit3 may reflect ‒ but not strongly drive ‒ ILD severity.
• Potential dual role as a diagnostic biomarker and future therapeutic target in RA-ILD pathogenesis.

## Introduction

Rheumatoid Arthritis (RA) is a chronic systemic inflammatory disease that can result in irreversible changes in the joints, leading to critical disability and poor quality of life.^1^ Studies have explored the association of RA and various indicators, such as inflammatory cytokines, autoantibodies, and destructive enzymes, to identify the therapeutic target of RA.^2^ Not only characterized by progressive and erosive destruction of the joints, but extra-articular diseases that occur in up to 50 % of patients with RA also make treatment difficult and worsen the prognosis of RA.^3^ Interstitial Lung Disease (ILD), the most common extra-articular complication of RA, clinically affects up to 10 % of patients with RA and subclinically affects up to 40 % of patients with RA. Furthermore, RA-ILD is a leading cause of death in patients with RA, leading to significant morbidity and mortality.^4^^,^^5^ Identification of the factors associated with RA-ILD, especially peripheral blood biomarkers, can help precisely manage and improve the prognosis of patients with RA.^6^

Slit guidance Ligand 3 (Slit3), a member of the axon guidance molecule superfamily, is a highly conserved secretory glycoprotein that is widely expressed in various tissues. By binding to their receptor Robos, Slits activate intracellular signaling pathways to mediate a series of biological activities, including axonal rejection, neuronal migration, organ development, reproductive regulation, tumor metastasis, angiogenesis, etc.^7^^,^^8^ It was reported that Slit3 can enhance monocyte migration in a concentration-dependent and chemoattractant-independent manner, thereby contributing to the recruitment of myeloid leukocytes to the inflamed peritoneum in vivo.^9^ Slit3 has also been implicated in bone metabolism, coordinating bone resorption and formation through osteoclast activity.^10^ Slit3 also showed the potential as a therapeutic target for fibrotic diseases. Previous studies have shown that Slit3 is highly expressed in cardiac fibroblasts, promotes their migration and differentiation, and increases the production of fibrillar collagen.^11^^,^^12^ SLIT3 expression level was positively correlated with the expression of fibrosis-related proteins in chronic liver disease, and SLIT3 deficiency alleviated TGF-β-induced hepatic stellate cell activation by inhibiting YAP activity.^13^ These data showed that Slit3 may be a key regulator of inflammatory response and ILD. However, the expression and clinical significance of Slit3 in RA have not been reported.

In this study, we first analyzed the expression of Slit3 in the peripheral blood of patients with RA and explored the clinical significance of Slit3. Especially, we measured its relationship with RA activity and detected its role as a serum biomarker for RA-ILD. We present this article in accordance with the Strengthening the Reporting of Observational Studies in Epidemiology reporting checklist.

## Methods

### Data sources

Between November 2022 and April 2024, patients with RA aged ≥16-years who fulfilled the 2010 American College of Rheumatology (ACR)/European League Against Rheumatism (EULAR) classification criteria for RA^14^ were included in the Department of Rheumatology of Foshan First People's Hospital, China. The exclusion criteria were as follows: overlapping with other autoimmune diseases (e.g. systemic lupus erythematosus, scleroderma, and dermatomyositis), serious infection, chronic liver disease, serious cardiovascular and cerebrovascular diseases, malignancy, pregnancy and ILD caused by other pulmonary diseases (e.g. infection and occupational exposure). Healthy control subjects were enrolled from the Physical Examination Center of the hospital. Healthy controls were excluded if they had any of the following: current infection, serious liver/cardiovascular/cerebrovascular disease, malignancy, pregnancy, severe mental disorders, or current use of medications such as NSAIDs, glucocorticoids, or antibiotics. The Ethics Committee of Foshan First People's Hospital approved the protocol of this study (ethics approval number FSEC-KS-2022–201), and all subjects signed informed consent forms before collecting clinical data. The study was conducted in accordance with the 1964 Declaration of Helsinki and its later amendments. This article was prepared in accordance with the STROBE (Strengthening the Reporting of Observational Studies in Epidemiology) guidelines.

### Clinical assessments

In this study, a total of 232 RA patients and 350 healthy controls were enrolled. The demographic and clinical data of patients, including age, gender, disease duration, disease activity, physical function, radiographic indicators, and medications, were collected from hospital records. Disease duration was divided into three categories: < 6-months (short), 6‒24 months (intermediate), and > 24-months (long). Health Assessment Questionnaire Disability Index (HAQ-DI) was used to assess physical activity function in eight categories (dressing, rising, eating, walking, hygiene, reaching, griping and activities) as described previously.^15^^,^^16^

Disease activity was assessed using the disease activity score in 28 joints with four variables, including C-Reactive Protein (DAS28-CRP) or erythrocyte sedimentation rate (DAS28-ESR), the Simplified Disease Activity Index (SDAI), and the Clinical Disease Activity Index (CDAI). Disease activity, measured based on DAS28-CRP, was divided into four categories: high disease activity (DAS28-CRP > 5.1), moderate disease activity (3.2 ≤ DAS28-CRP ≤ 5.1), low disease activity (2.6 ≤ DAS28-CRP < 3.2), and remission (DAS28-CRP < 2.6).^17^ Conventional radiographs of bilateral hands and wrists (anteroposterior view) were assessed in 120 RA patients with the Sharp/van der Heijde modified score, as described previously. A total of 16 areas for joint erosion and 15 for Joint-Space Narrowing (JSN) of the hands were assessed in each hand/wrist. The maximum score per single joint for erosion is 5, and for JSN is 4, with the sum of erosion (0‒160) and JSN (0‒120) subscores constituting the modified Total Sharp Score (mTSS, 0‒280).^15^^,^^18^ ILD was diagnosed based on fibrotic abnormalities, particularly traction bronchiectasis or honeycombing, in >10 % of lung parenchyma on High-Resolution Computed Tomography (HRCT) findings, and with no evidence of other diagnoses.^19^ The severity and extent of ILD were assessed by percentage of the affected lung tissue in HRCT, 6-minute walk test and Pulmonary Function Testing (PFT), including measurements of Forced Expiratory Volume in one second (FEV1), Forced Vital Capacity (FVC), FEV1/FVC ratio, as well as diffusion of the lungs for carbon monoxide (DLco).^20^^,^^21^ The study was conducted in accordance with the 1964 Declaration of Helsinki and its later amendments.

### Measurement of the serum levels of slit3

The serum samples of patients with RA and healthy controls were collected after overnight fasting and stored at −80 °C. The serum level of Slit3 was measured using a commercially available Enzyme-Linked Immunosorbent Assay (ELISA) kit (NeoBioscience Technology Co., Ltd., OKDD00532) following the manufacturer’s instructions.

### Statistical analysis

Statistical analyses were conducted using SPSS software (version 25.0). Continuous variables are presented as mean ± Standard Deviations (SD) or median and Interquartile Range (IQR) based on data distribution. We used the Kolmogorov-Smirnov test to measure the normality of the data. Categorical variables are presented as frequencies and percentages. Correlation analyses were performed using Spearman's rank correlation coefficient, as the data were non-parametrically distributed.

A 1:1 individually matched case-case comparative study was conducted to characterize epidemiological characteristics of patients with RA and healthy controls. RA patients were initially stratified by gender, followed by matching individual patients within each gender stratum based on age with one healthy control. This two-step matching strategy aimed to balance the heterogeneity of age and gender across RA patients and healthy controls.

The Mann-Whitney test was employed to compare the differences in continuous variables between the two groups. The Chi-Square test or Fisher's exact test was used to compare categorical variables in the two groups. Univariate and multivariate logistic regression analyses were used to identify the potential associated factors with RA-ILD. All tests were two-tailed and *p* < 0.05 was considered the threshold of statistical significance.

## Results

### Baseline characteristics of the included participants

Finally, we included 232 patients with RA and 350 healthy controls in this study (Table 1). None of the continuous variables studied in this study were normally distributed. In the RA group, the median age was 62.0 (54.3, 71.0) years, with 73.7 % of participants being female. The median disease duration was 48 (12, 120) months, 15.9 % of participants had short disease duration (< 6-months), and 59.5 % of participants had long disease duration (> 24-months). Based on the DAS28-CRP, 27.6 %, 43.1 %, 11.6 %, and 17.7 % of patients were in the high, moderate, low disease activity, and remission groups, respectively. In total, 20.3 % of patients did not receive glucocorticoids or conventional synthetic Disease-Modifying Anti-Rheumatic Drugs (DMARDs) for at least six months before enrolment (treatment-naïve patients).

**Table 1:** Baseline characteristics of RA patients and healthy controls.

Characteristics	Healthy controls (*n* = 350)	Healthy controls (*n* = 232) after matched	All RA patients (*n* = 232) after matched	p^a^ after matched
Age, years, median (IQR)	50.5 (39.0, 62.0)	61.0 (57.0, 66.0)	62.0 (54.3, 71.0)	0.457
Female, n ( %)	211 (60.3)	171 (73.7)	171 (73.7)	1.00
Disease duration, month, median (IQR)	‒		48 (12, 120)	
Positive RF, n ( %)	‒		164 (70.7)	
Positive ACPA, n ( %)	‒		167 (72.0)	
Core disease activity indicators				
28TJC, median (IQR)	‒		5.5 (1, 17)	
28SJC, median (IQR)	‒		4 (1, 14)	
PtGA, cm, median (IQR)	‒		6 (5, 7)	
PrGA, cm, median (IQR)	‒		5 (4, 6)	
Pain VAS, cm, median (IQR)	‒		5 (4, 5)	
ESR, mm/h, median (IQR)	‒		44.5 (25.3, 66.0)	
CRP, mg/L, median (IQR)	‒		19.9 (4.9, 57.4)	
DAS28-CRP, median (IQR)	‒		3.9 (2.9, 5.2)	
DAS28-ESR, median (IQR)	‒		4.5 (3.5, 5.8)	
SDAI, median (IQR)	‒		25.0 (15.5, 45.0)	
CDAI, median (IQR)	‒		23 (12, 40)	
Functional indicator				
HAQ-DI, median (IQR)	‒		1.00 (0.63, 1.25)	
Radiographic indicators				
mTSS, median (IQR)	‒		16 (5, 29)	
JSN subscore, median (IQR)	‒		9 (3, 17)	
JE subscore, median (IQR)	‒		6 (2, 11)	
Previous medications				
Treatment naïve^b^, n ( %)	‒		47 (20.3)	
Glucocorticoid, n ( %)	‒		114 (49.1)	
csDMARDs, n ( %)	‒		138 (59.5)	
Biologic agents, n ( %)	‒		26 (11.2)	

Data are shown as median (interquartile range) or n ( %).

ACPA, Anti-cyclic Citrullinated Peptide Antibody; CRP, C-Reactive Protein; CDAI, Clinical Disease Activity Index; DAS28-CRP, Disease Activity Score in 28-joints including CRP; ESR, Erythrocyte Sedimentation Rate; HAQ-DI, Stanford Health Assessment Questionnaire Disability Index; JSN, Joint Space Narrowing; JE, Joint Erosion; mTSS, Modified total Sharp Score; PtGA, Patient Global Assessment of disease activity; PrGA, Provider Global Assessment of disease activity; Pain VAS, Pain Visual Analogue Scale; RF, Rheumatoid Factor; SDAI, Simplified Disease Activity Index; SJC28, 28-joint swollen joint count; TJC28, 28-joint tender joint count.

Treatment naive^b^, without previous corticosteroids or DMARDs therapy for at least 6-months before enrollment.

a Compared between healthy controls and RA patients by Mann-Whitney test or Chi-square test.

Before matching, in the healthy control group (*n* = 350), the median age was 50.5 (39.0, 62.0) years, which was younger than that of patients in the RA group. Healthy controls also showed a lower prevalence of females (60.3 % vs. 73.7 %) than RA patients (all *p* < 0.001). Then an individual matching was conducted to balance the effects of age and gender between two groups, and a total of 232 healthy control subjects were age- and gender-matched to the RA patients individually at a ratio of 1 to 1 to further compare the serum level of Slit3. In the matched healthy control group (*n* = 232), 73.7 % of participants were females, and the median age was 61.0 (57.0, 66.0) years, which showed no difference with the RA group (*p* = 0.457, Table 1).

### The serum level of Slit3 in patients with RA

**Fig. 1:**
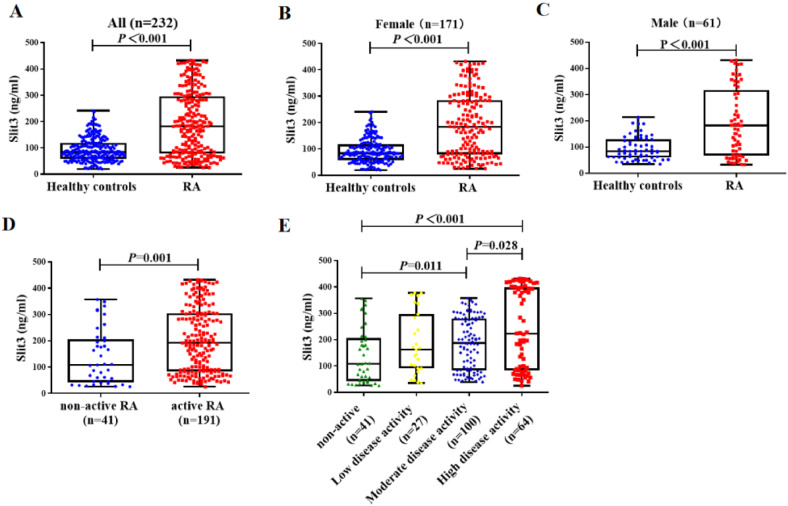
Serum Slit3 level in RA patients and matched healthy controls. (A, B, C) Baseline serum Slit3 level in RA patients and matched healthy controls; (D‒E) Serum Slit3 level in non-active RA (DAS28-CRP < 2.6) and active RA patients (DAS28-CRP ≥ 2.6).

Compared to matched healthy subjects, patients with RA had a higher serum level of Slit3 (182.0 [78.7‒296.6] ng/mL vs. 104.1 [66.5‒191.1] ng/mL, *p* < 0.001, Fig. 1A). Furthermore, gender stratification analysis showed that both females and males with RA had higher serum levels of Slit3 (female: 183.7 [79.4‒284.6] ng/mL vs. 103.0 [68.1‒192.7] ng/mL, *p* < 0.001, Fig. 1B; male: 181.7 [67.3‒317.0] ng/mL vs. 107.3 [62.6‒187.5] ng/mL, *p* < 0.001, Fig. 1C). Furtherly, active RA patients (DAS28-CRP ≥ 2.6) showed higher level of serum Slit3 than those non-active RA (DAS28-CRP < 2.6) (191.7 [84.1‒304.1] ng/mL vs. 108.0 [42.5‒205.6] ng/mL, *p* = 0.001]. In Active RA patients, those with high active disease showed highest level of serum Slit3, and showed statistical significance than non-active RA and those with moderate disease activity (222.3 [83.9‒398.5] ng/mL vs. 108.0 [42.5‒205.6] ng/mL, *p* < 0.001; 222.3 [83.9‒398.5] ng/mL vs. 186.7 [83.7‒280.7] ng/mL, *p* = 0.028, Fig. 1D‒E].

### Comparison of disease characteristics between low and high serum Slit3 subgroups

While there is no universally established clinical cut-off for serum Slit3, and the level of serum Slit3 is not symmetrically distributed in the present study. Based on the median serum level of Slit3 (182.0 ng/mL), patients with RA were stratified into two subgroups, including the low Slit3 group (*n* = 116) and the high Slit3 group (*n* = 116). Patients with RA were older in the high Slit3 group (median 65 vs. 59), were more likely to be positive for Rheumatoid Factor (RF) (76.7 % vs. 64.7 %) and Anti-cyclic Citrullinated Peptide Antibody (APCA) (80.2 % vs. 63.8 %) and had a higher level of disease activity indicators, including ESR (median 56 vs. 36), CRP (median 29.6 vs. 10.6), DAS28-CRP (median 4.2 vs. 3.6), DAS28-ESR (median 4.8 vs. 4.2) and SDAI (median 31.5 vs. 23.1, all *p* < 0.05, Table 2, Table 3). Among the 120 RA patients assessed with the Sharp/van der Heijde modified score, 46 patients were classified into the low Slit3 group and 66 patients were classified into the high Slit3 group. There were no differences in JSN subscore, JE subscore, mTSS and the proportion of joint erosion between the two groups (all *p* > 0.05). The proportion of joint erosion in the high Slit3 group was higher than that in the low Slit3 group (95.5 % vs. 84.8 %, *p* = 0.051).

**Table 2:** Characteristics (continuous variables) of RA patients in serum Slit3 subgroups.

	Low Slit3 group (*n* = 116)	High Slit3 group (*n* = 116)	p
**Age, years**	59 (53, 67)	65 (56, 73)	**0.008**
**Disease duration, mo**	48 (12, 120)	48 (12, 120)	0.864
**Core disease activity indicators**			
28TJC	4 (1,16)	8 (2, 18)	0.056
28SJC	2 (0,16)	5 (1, 14)	0.214
PtGA	5 (4,6)	6 (5, 7)	0.057
PrGA	5 (4,6)	5 (4,6)	0.017
Pain VAS	5 (3,5)	5 (4, 6)	0.058
ESR (mm/h)	36.0 (19.0, 57.0)	56.0 (33.0, 72.0)	**<0.001**
CRP (mg/L)	10.6 (2.9, 40.9)	29.6 (7.5, 71.0)	**<0.001**
DAS28-CRP	3.6 (2.6, 5.0)	4.2 (3.5, 5.4)	**0.003**
DAS28-ESR	4.2 (3.2, 5.4)	4.8 (3.9, 5.9)	**0.003**
SDAI	23.1 (12.9, 41.0)	31.5 (19.0, 46.7)	**0.016**
CDAI	18 (11, 38)	29 (14, 41)	0.068
**Functional assessment**			
HAQ-DI	0.8 (0.5, 1.1)	1.1 (0.6, 1.3)	**0.001**
**Radiographic assessments**			
mTSS	15 (5, 31)	16 (5, 28)	0.995
JSN subscore	11 (2, 17)	9 (3, 16)	0.776
JE subscore	5 (2, 12)	6 (2, 11)	0.623

Data are shown as median (interquartile range).

*Compared between RA patients in low slit3 subgroup and high slit3 subgroup by Mann-Whitney test. Significant differences are shown in bold.

CRP, C-Reactive Protein; CDAI, Clinical Disease Activity Index; DAS28-CRP, Disease Activity Score in 28-joints including CRP; ESR, Erythrocyte Sedimentation Rate; HAQ-DI, Stanford Health Assessment Questionnaire Disability Index; JSN, Joint Space Narrowing; JE, Joint Erosion; mTSS, Modified Total Sharp Score; PtGA, Patient global Assessment of disease Activity; PrGA, Provider Global Assessment of disease Activity; Pain VAS, Pain Visual Analogue Scale; RF, Rheumatoid Factor; SDAI, Simplified Disease Activity Index; SJC28, 28-Joint Swollen Joint Count; TJC28, 28-joint Tender Joint Count.

**Table 3:** Characteristics (categorical variables) of RA patients in serum Slit3 subgroups.

	Low Slit3 group (*n* = 116)	High Slit3 group (*n* = 116)	p
**Female**	87 (75.0)	84 (72.4)	0.655
**Positive RF**	75 (64.7)	89 (76.7)	**0.043**
**Positive ACPA**	74 (63.8)	93 (80.2)	**0.005**
**JE**	39 (84.8)	63 (95.5)	0.051
**Previous medications**			
Treatment-naive^a^	27 (23.3)	20 (17.2)	0.253
Corticosteroids	51 (44.0)	63 (54.3)	0.115
csDMARDs	65 (56.0)	73 (62.9)	0.285
Biologic agents	15 (12.9)	11 (9.5)	0.405

Data are shown as n ( %).

*Compared between RA patients in low slit3 subgroup and high slit3 subgroup by Chi-square test. Significant differences are shown in bold.

ACPA, Anti-cyclic Citrullinated Peptide Antibody; RF, Rheumatoid Factor; JE, Joint Erosion.

Treatment naive^a^, without previous corticosteroids or DMARDs therapy for at least 6-months before enrollment.

**Fig. 2:**
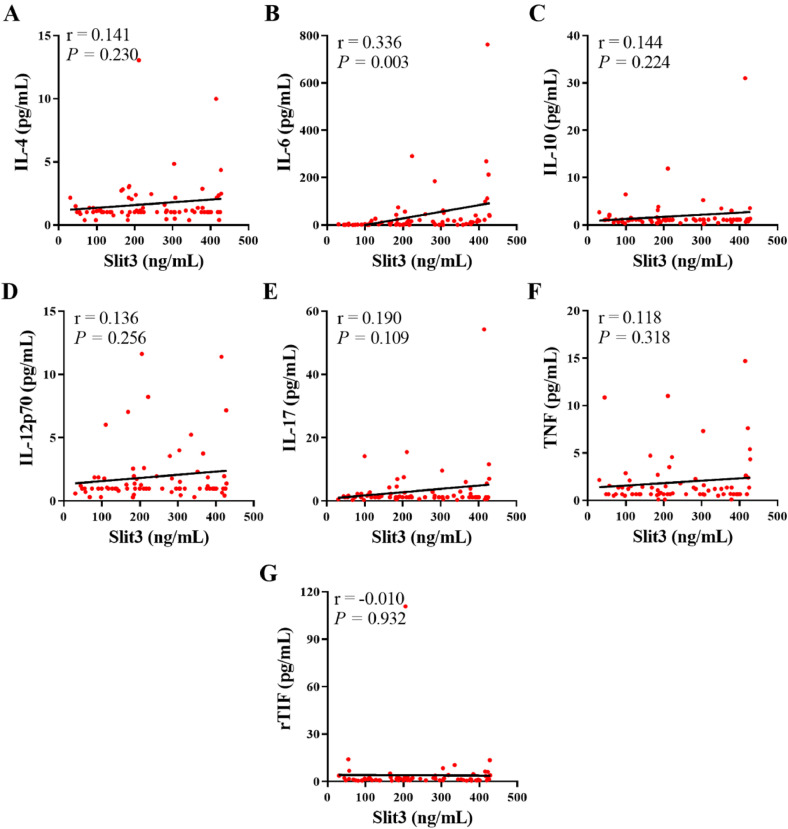
Correlation between serum Slit3 with different serum cytokine levels. (A) IL-4; (B) IL-6; (C) IL-10; (D) IL-12p70; (E) IL-17; (F) TNF; (G) rTIF.

Furthermore, the correlation between the serum levels of Slit3 and the serum levels of different serum cytokines was measured in 75 patients with RA. The serum level of Slit3 was significantly and positively correlated with the serum level of the pro-inflammatory cytokine interleukin-6 (IL-6) (*r* = 0.336, *p* = 0.003, Fig. 2A‒G), but not with the serum level of other cytokines, including IL-4, IL-10, IL-12p70, IL-17, Tumor Necrosis Factor (TNF), and r-Transcriptional Intermediate Factor (rTIF).

### The characteristics of patients with RA-ILD

Among 232 patients with RA, 45 (19.4 %) patients with RA had ILD. Compared to those without ILD (RA-no-ILD), RA patients with ILD were older (median 66 vs. 62), were less likely to be female (51.1 % vs. 79.1 %), were more likely to be positive for RF (88.9 % vs. 66.3 %) and ACPA (91.1 % vs. 67.4 %), and had higher levels of ESR (median 59 vs. 42), HAQ-DI (median 1.1 vs. 0.9), and Slit3 (median 259.5 vs. 168.8, all *p* < 0.05, Table 4).

**Table 4:** Comparison of characteristics between RA-ILD and RA-no-ILD.

	RA-ILD (*n* = 45)	RA-no-ILD (*n* = 187)	p
**Age, years, median (IQR)**	66 (58, 73)	62 (54, 69)	**0.025**
**Female, n ( %)**	23 (51.1)	148 (79.1)	**<0.001**
**Disease duration, mo, median (IQR)**	84 (24, 144)	36 (7, 120)	**0.010**
**Positive RF, n ( %)**	40 (88.9)	124 (66.3)	**0.003**
**Positive ACPA, n ( %)**	41 (91.1)	126 (67.4)	**0.001**
**Core disease activity indicators**			
28TJC, median (IQR)	6 (1, 21)	5 (1, 15)	0.441
28SJC, median (IQR)	5 (0, 18)	4 (1, 14)	0.895
PtGA, median (IQR)	6 (5, 7)	6 (5, 7)	0.281
PrGA, median (IQR)	5 (4, 6)	5 (4, 6)	0.361
Pain VAS, median (IQR)	5 (3, 6)	5 (4, 5)	0.733
ESR (mm/h), median (IQR)	59.0 (40.5, 73.0)	42.0 (24.0, 64.0)	**0.010**
CRP (mg/L), median (IQR)	24.7 (6.5, 69.7)	16.4 (4.6, 54.5)	0.237
DAS28-CRP, median (IQR)	4.0 (3.2, 5.6)	3.9 (2.8, 5.2)	0.393
DAS28-ESR, median (IQR)	4.7 (3.7, 6.2)	4.5 (3.4, 5.7)	0.303
SDAI, median (IQR)	32.2 (18.7, 51.7)	24.1 (15.2, 42.7)	0.232
CDAI, median (IQR)	28 (13, 45)	22 (12, 38)	0.322
**Functional assessment**			
HAQ-DI, median (IQR)	1.1 (0.7, 1.4)	0.9 (0.5, 1.3)	**0.034**
**Radiographic assessments**			
mTSS, median (IQR)	20 (13, 32)	13 (4, 28)	0.144
JSN subscore, median (IQR)	13 (6, 22)	9 (2, 16)	0.144
JE subscore, median (IQR)	8 (5, 13)	5 (2, 11)	0.109
**Previous medications**			
Treatment-naive^a^, n ( %)	5 (11.1)	42 (22.5)	0.089
Glucorticosteroids, n ( %)	24 (53.3)	90 (48.1)	0.531
csDMARDs, n ( %)	32 (71.1)	106 (56.7)	0.077
Biologic agents, n ( %)	5 (11.1)	21 (11.2)	0.982
Serum Slit3, median (IQR)	259.5 (143.5, 362.3)	168.8 (70.0, 281.3)	**0.001**

Data are shown as median (interquartile range) or n ( %).

*Compared between RA-ILD subgroup and RA-no-ILD subgroup by Mann-Whitney test or Chi-Square test. Significant differences are shown in bold.

ACPA, Anti-Cyclic Citrullinated Peptide Antibody; CRP, C-Reactive Protein; CDAI, Clinical Disease Activity Index; DAS28-CRP, Disease Activity Score in 28-joints including CRP; ESR, Erythrocyte Sedimentation Rate; HAQ-DI, Stanford Health Assessment Questionnaire Disability Index; JSN, Joint Space Narrowing; JE, Joint Erosion; mTSS, Modified Total Sharp Score; PtGA, Patient Global Assessment of disease Activity; PrGA, Provider Global Assessment of disease Activity; Pain VAS, Pain Visual Analogue Scale; RF, Rheumatoid Factor; SDAI, Simplified Disease Activity Index; SJC28, 28-joint Swollen Joint Count; TJC28, 28-joint Tender Joint Count.

Treatment naive^a^, without previous corticosteroids or DMARDs therapy for at least 6-months before enrollmen.

**Fig. 3:**
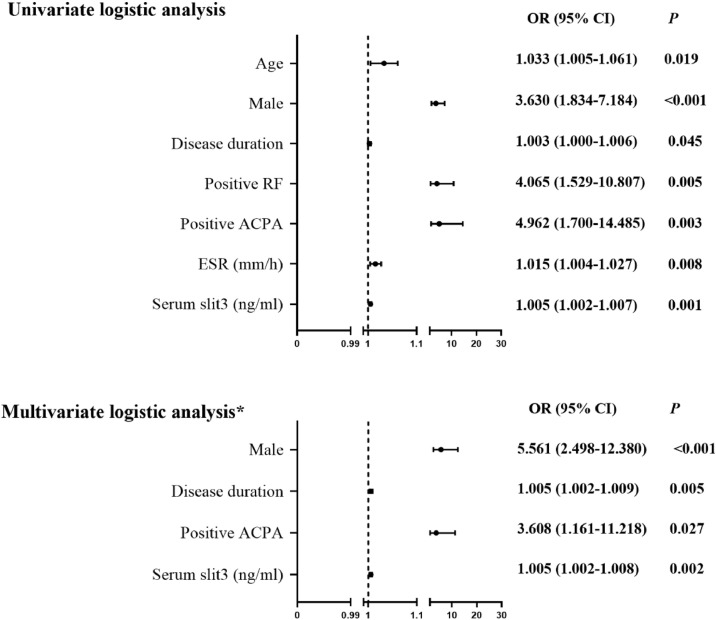
Logistic regression analysis of the relevant characteristics of RA-ILD. ACPA, Anti-Cyclic Citrullinated Peptide Antibody; ESR, Erythrocyte Sedimentation Rate; RF, Rheumatoid Factor; OR, Odds Ratio; CI, Confidence Interval. *Stepwise multivariate logistic regression analysis.

Univariate logistic regression analysis showed that RA-ILD was positively associated with high serum levels of Slit3 (Odds Ratio [OR = 1.005], 95 % CI 1.002‒1.007), male gender (OR = 3.630, 95 % CI 1.834‒7.184), long disease duration (OR = 1.003, 95 % CI 1.000‒1.006), positive RF status (OR = 4.065, 95 % CI 1.529‒10.807), positive ACPA status (OR = 4.962, 95 % CI 1.700‒14.485), and high ESR levels (OR = 1.015, 95 % CI 1.004‒1.027, all *p* < 0.05]. Further stepwise multivariate logistic regression analysis, including the significant indicators mentioned above, showed that high serum Slit3 (OR = 1.005, 95 % CI 1.002‒1.008), male gender (OR = 5.561, 95 % CI 2.498‒12.380), long disease duration (OR = 1.005, 95 % CI 1.002‒1.009), and positive ACPA status (OR = 3.608, 95 % CI 1.161‒11.218, all *p* < 0.05] were independently associated with RA-ILD (Fig. 3). We performed an additional forced-entry multivariable logistic regression including all candidate variables. Prior use of glucocorticosteroids and cDMARDs was not associated with RA-ILD (Table S1), confirming that their exclusion from the stepwise model did not affect the validity of the identified independent risk factors.

As an exploratory analysis limited by the small number of patients with available data, we assessed the influence of serum Slit3 on the severity and extent of ILD (Fig. S1). The serum level of Slit3 was positively correlated with the percentage of the affected lung tissue in HRCT (*r* = 0.372, *p* = 0.012). However, the serum level of Slit3 showed no correlation with the 6-min walk distance (*r* = −0.495, *p* = 0.072) as only 14 of 45 RA patients with ILD underwent the 6-minute walk test. PFT were conducted on 25 RA patients had ILD, and the results showed that the serum level of Slit3 was negatively but not significantly correlated with FEV1/FVC (*r* = −0.395, *p* = 0.051) and DLco (*r* = −0.428, *p* = 0.067), likely due to the small sample size (*n* = 25 for PFTs, *n* = 14 for 6-minute walk test) increasing the risk of Type II error. These findings are hypothesis-generating and require validation in larger cohorts. Therefore, these exploratory findings should be interpreted with caution due to the limited sample size and require validation in larger cohorts.

## Discussion

In this study, we investigated the relationship between serum Slit3 and disease characteristics in patients with RA. Patients with RA and high Slit3 levels showed higher disease activity and a higher proportion of ILD, and Slit3 served as an independent risk factor for RA-ILD. These findings suggest that serum Slit3 is associated with higher disease activity and RA-ILD, indicating its potential as a biomarker. The observed correlation does not establish causality, reinforcing the need for longitudinal studies. Its functional role in RA-ILD pathogenesis remains to be elucidated through further mechanistic studies.

Slit3 is a highly conserved secreted glycoprotein in many organs. It is highly expressed in human fibroblasts, tenocytes, adipocytes, mesenchymal cells, and smooth muscle cells.^7^ In the majority of tissues, Slit3 can bind to Robo receptors to transmit cellular signals and regulate various life activities.^7^^,^^8^ Previous studies have shown that Slit3 acts as an angiogenic factor, stimulating endothelial cell proliferation, motility, chemotaxis, and formation of an endothelial vascular network by interacting with Robo4.^22^ Silencing Slit3 promoted the proliferation, migration, and invasion of lung adenocarcinoma, and treatment with Slit3 inhibited the migration of malignant melanoma cells.^23^^,^^24^ Recent studies have shown that osteoclast-derived Slit3 plays an osteoprotective role by synchronously stimulating bone formation and suppressing bone resorption through Robo receptors, making it a potential therapeutic target for metabolic bone disorders.^25^^,^^26^ Another study showed that the mRNA levels of Slit2 and Slit3 are higher than the mRNA levels of Slit1 in RA Synovial Fibroblasts (RA-SF). Compared to RA-SF and normal SF, RA-SF showed a lower expression level of Slit3, and Slit3 inhibited Robo3-induced migration of RA-SF.^27^ In this study, compared to healthy controls, both women and men with RA showed a higher serum level of Slit3. More studies are needed to explore the main source of Slit3 in RA.

Previous studies have identified the link between Slit3 and inflammation. Stimulation with Slit3 increased the spontaneous and chemoattractant-induced migration of primary monocytes in vitro and promoted myeloid cell recruitment after peritoneal inflammation in vivo.^9^ The total amounts and concentrations of Slit3 were significantly higher in periodontitis than in healthy tissue, and a significant positive correlation was observed between Slit3 level in the gingival crevicular fluid and radiographic bone loss.^28^ Elevated levels of Slit3 in amniotic and myometrium cells lead to the rupture of the fetal membrane and preterm birth by promoting gene expression and the release of IL-1β-induced pro-inflammatory cytokines (IL-6 and IL-8) and matrix metalloproteinase-9.^29^ By dividing 490 patients with lung adenocarcinoma in the Cancer Genome Atlas database into the high-inflammatory and low-inflammatory groups, Slit3 expression was upregulated in the high-inflammatory index group, and patients with high expression of Slit3 showed a good prognosis.^30^ In the present study, patients with RA in the high serum slit3 group showed higher disease activity indicators, including ESR, CRP, DAS28-CRP, DAS28-ESR, and SDAI. Slit3 also showed a significant correlation with the pro-inflammatory cytokine IL-6. All these findings indicated the pro-inflammatory role of Slit3 in RA.

RA-ILD is a major cause of death in patients with RA, leading to significant morbidity and mortality. While it can be the initial manifestation in 10 % to 20 % of patients, most cases of RA-ILD are diagnosed within the first five years after the initial diagnosis of RA.^31^ The assessment of risk factors is crucial for RA-ILD due to its importance in mortality and treatment. Studies have shown that some significant Human Leukocyte Antigen (HLA) variations, including HLA-DRB1, HLA-DR4, and HLA-B40, can contribute to the development of ILD in patients with RA. Antibodies (e.g., RF and ACPA), lung epithelial-related proteins (e.g., Lungen-6 and Surfactant Protein D [SP-D]), cytokines (e.g., CCL18 and IL-6), and other molecules (e.g., MMP-7, ESR, and CRP) are also important biomarkers of RA-ILD.^32^^,^^33^ A study showed that compared to patients with RA who do not have ILD, those with ILD were more likely to be men or older and have higher DAS28-ESR levels.^34^ Similarly, in the present study, male gender, long disease duration, and positive ACPA status were independent risk factors of RA-ILD. In addition, the exploratory nature of the ILD severity analysis, limited by small sample sizes, precludes definitive conclusions and highlights the need for larger studies to confirm these preliminary findings.

Apart from the inflammatory cascade, fibroblast proliferation and activation are the main drivers of ILD.^35^ Analysis of Gene Expression Omnibus data showed that Slit3 is upregulated in the liver tissue of people with fibrosing non-alcoholic steatohepatitis, and Slit3 expression levels were positively correlated with fibrosis-related proteins. Human umbilical cord-derived mesenchymal stem cells can alleviate liver fibrosis by inhibiting the activation of hepatic stellate cells through the miR-148a-5p/SLIT3 pathway.^13^^,^^36^ Another study showed that the Slit3 expression level was increased in Ang II-induced mouse models and cardiac fibroblasts and promoted cardiac fibrosis and fibroblast differentiation via the RhoA/ROCK1 signaling pathway.^12^ These data imply the importance of Slit3 as a potential therapeutic target in tissue fibrosis.^37^ However, another study showed that Slit2 injection in mice can inhibit bleomycin-induced lung fibrosis. In lung tissues from patients with pulmonary fibrosis and relatively normal lung function, Slit2 has a widespread distribution. In contrast, Slit2 is lowly expressed in the fibrotic lesions in patients with advanced disease.^38^ In the present study, the high serum level of Slit3 was an independent risk factor for RA-ILD. The mechanism by which Slit3 is involved in RA-ILD and lung fibrosis is worth further exploration.

The present study also had some limitations. Firstly, it was a cross-sectional and retrospective study. Risk factors and outcome measures were assessed at the same time and causality could not be inferred. Future large-scale multi-center prospective studies on patients with RA are needed to address these limitations and make potentially novel discoveries. Secondly, due to the small sample size, we could not analyze the factors associated with different subtypes of ILD. Thirdly, observed Spearman's correlation coefficients between serum Slit3 and IL-6 (*r* = 0.336, *p* = 0.003) as well as with the percentage of affected lung tissue on HRCT (*r* = 0.372, *p* = 0.012) indicate weak-to-moderate positive associations. These effect sizes suggest that, while statistically significant, the strength of these linear relationships is limited, and they do not imply causation. Larger studies are needed to confirm the relationship of serum Slit3 with RA-ILD subtypes and the clinical relevance of these associations and to explore potential non-linear or indirect relationships. Thirdly, although prior exposure to glucocorticoids and conventional synthetic DMARDs was not independently associated with RA-ILD in this analysis, we cannot fully rule out a possible influence of these medications on serum Slit3 concentrations due to the design of the study. Finally, the observed correlation between serum Slit3 and the extent of lung involvement on HRCT must be regarded as exploratory, given that functional assessments (6MWT and PFT) were available in only a minority of RA-ILD patients. Larger prospective studies with comprehensive pulmonary evaluations are needed to clarify whether Slit3 reflects ILD severity or progression. In summary, future studies should explore correlations between serum Slit3 and additional ILD parameters, including Forced Vital Capacity (FVC), 6-minute walk test, volumetric HRCT, and oxygenation measures (e.g., oxygen saturation and PO_2_), to further evaluate Slit3’s role in ILD severity and progression.

## Conclusion

In conclusion, we found that elevated serum Slit3 was associated with higher disease activity and inflammation in RA, and was an independent risk factor for RA-ILD. Slit3 shows promise as a candidate serum biomarker and warrants further investigation as a potential therapeutic target for RA-ILD.

## Data availability

The data are available from the corresponding author upon reasonable request.

## Compliance with ethical standards

All human studies were approved by the ethics committee on human research of Foshan First People's Hospital (n° FSEC-KS-2022–201). All procedures were conducted in accordance with the ethical standards defined in the 1964 Declaration of Helsinki and its later amendments. All persons gave their informed consent before their inclusion in this study.

## Clinical trial number

Not Applicable.

## Authors’ contributions

Xuepei Zhang: Conceptualization; methodology; formal analysis; writing-original draft preparation. Maohua Shi: Data curation; investigation; resources; funding acquisition. Dongmei Guo: Data curation; investigation. Yangtao Yu: Validation; writing-reviewing and editing. Hongwei Zhang: Validation, writing-reviewing and editing. Guoqiang Chen: Conceptualization; supervision. All authors studied and approved the final manuscript for publication.

## Funding

This study was supported by the 10.13039/501100001809National Natural Science Foundation of China (grant no 82001742) from Maohua Shi, and Medical Science and Technology Research Fund of Guangdong Province (grant no B2023193) from Xuepei Zhang.

## Conflicts of interest

The authors declare no conflicts of interest.

## Appendix Supplementary materials

Image, application 1
