# Supplementary material for: Elevated serum Slit3 independently predicts disease activity and interstitial lung disease in rheumatoid arthritis
Source: Clinics (Sao Paulo). 2026 Apr 8;81:100935. doi: 10.1016/j.clinsp.2026.100935 (PMC13091409; doi:10.1016/j.clinsp.2026.100935)

**CLINICS-D-25-00448_Supplementary Material**

**Table S1** Logistic regression analysis of the relevant characteristics of RA-ILD in patients.

| **Characteristics** | **Univariate analysis** | | **Multivariate analysis^a^** | |
| --- | --- | --- | --- | --- |
|  | **OR (95% CI)** | **p** | **OR (95% CI)** | **p** |
| Treat-naive | 0.432 (0.160‒1.163) | 0.097 | 0.384 (0.092‒1.602) | 0.189 |
| Glucorticosteroids | 1.232 (0.642‒2.364) | 0.531 | 0.682 (0.281‒1.659) | 0.399 |
| csDMARDs | 1.881 (0.928‒3.813) | 0.080 | 1.130 (0.387‒3.298) | 0.823 |
| Age | **1.033 (1.005‒1.061)** | **0.019** | 1.016 (0.983‒1.051) | 0.343 |
| Male | **3.630 (1.834‒7.184)** | **<0.001** | **5.730 (2.327‒14.106)** | **<0.001** |
| Disease duration | **1.003 (1.000‒1.006)** | **0.045** | **1.005 (1.001‒1.009)** | **0.027** |
| Positive RF | **4.065 (1.529‒10.807)** | **0.005** | 2.290 (0.617‒8.499) | 0.216 |
| Positive ACPA | **4.962 (1.700‒14.485)** | **0.003** | 2.428 (0.637‒9.257) | 0.194 |
| 28TJC | 1.021 (0.987‒1.057) | 0.222 | **1.131 (1.022‒1.251)** | **0.017** |
| 28SJC | 1.011 (0.975‒1.048) | 0.558 | 1.055 (0.967‒1.151) | 0.230 |
| PtGA | 1.083 (0.890‒1.319) | 0.426 | 0.963 (0.473‒1.963) | 0.918 |
| PrGA | 1.099 (0.896‒1.348) | 0.363 | 1.302 (0.592‒2.865) | 0.512 |
| Pain VAS | 1.004 (0.849‒1.187) | 0.960 | 0.997 (0.660‒1.508) | 0.991 |
| ESR | **1.015 (1.004‒1.027)** | **0.008** | 1.015 (0.998‒1.031) | 0.080 |
| CRP | 1.006 (1.000‒1.013) | 0.056 | 1.011 (0.999‒1.023) | 0.079 |
| DAS28-CRP | 1.111 (0.897‒1.376) | 0.333 | 0.295 (0.086‒1.016) | 0.053 |
| Serum Slit3 | **1.005 (1.002‒1.007)** | **0.001** | **1.004 (1.001‒1.007)** | **0.042** |

^a^ Forced-entry multivariable logistic regression.

**Figure S1** The relationship between Slit3 and ILD severity assessed with olumetric HRCT chest “percentage of the affected lung tissue”, 6-minute walk test and PET. PET, Pulmonary function Testing; FEV1, Forced Expiratory Volume in one second; FVC, Forced Vital Capacity; DLco, Diffusion of the Lungs for carbon monoxide.


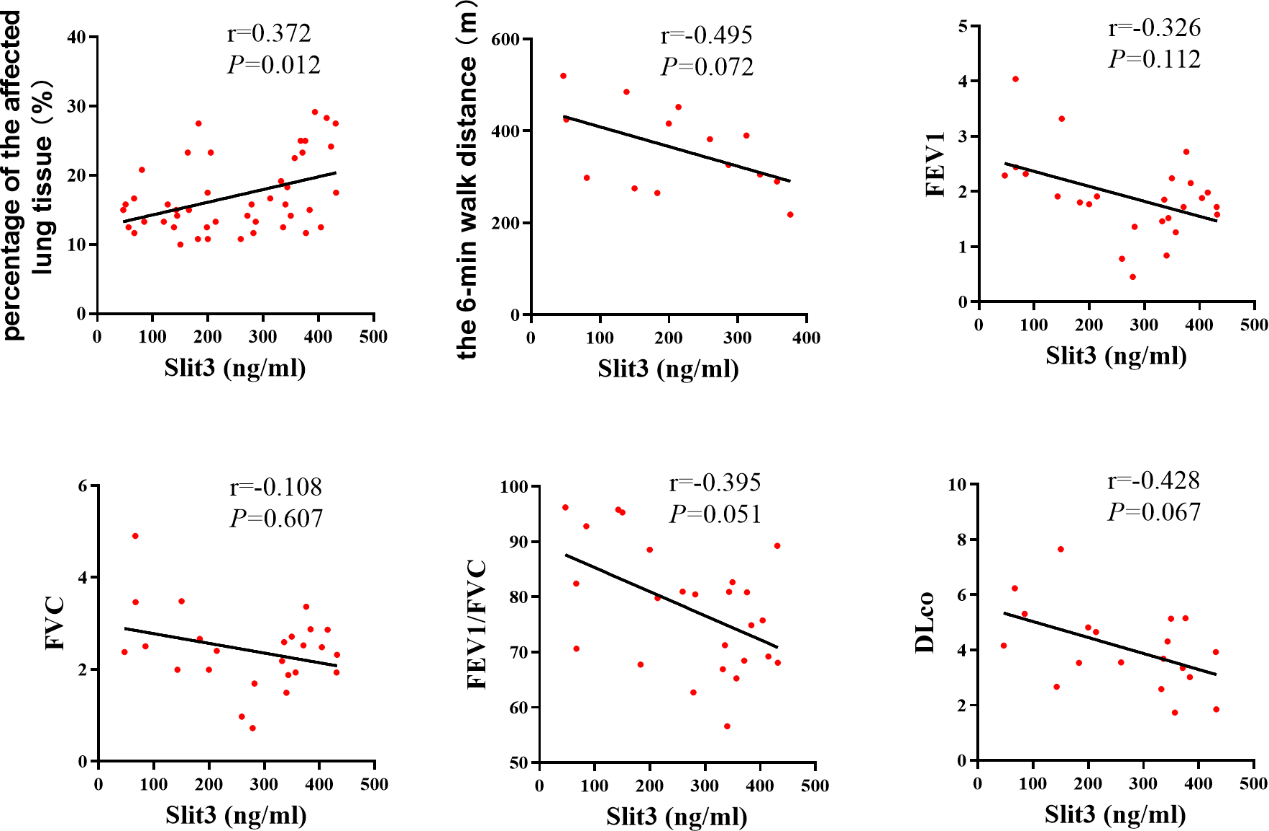

Supplement: Supplementary file 1 [file mmc1.docx]
